# Vibrating Flexoelectric Micro-Beams as Angular Rate Sensors

**DOI:** 10.3390/mi13081243

**Published:** 2022-08-02

**Authors:** Yilin Qu, Feng Jin, Jiashi Yang

**Affiliations:** 1 State Key Laboratory for Strength and Vibration of Mechanical Structures, Xi’an Jiaotong University, Xi’an 710049, China; 18229058666@163.com; 2 Department of Mechanical and Materials Engineering, University of Nebraska-Lincoln, Lincoln, NE 68588-0526, USA

**Keywords:** gyroscope, flexoelectric, beam, vibration

## Abstract

We studied flexoelectrically excited/detected bending vibrations in perpendicular directions of a micro-beam spinning about its axis. A set of one-dimensional equations was derived and used in a theoretical analysis. It is shown that the Coriolis effect associated with the spin produces an electrical output proportional to the angular rate of the spin when it is small. Thus, the beam can be used as a gyroscope for angular rate sensing. Compared to conventional piezoelectric beam gyroscopes, the flexoelectric beam proposed and analyzed has a simpler structure.

## 1. Introduction

Gyroscopes are key components for motion sensing. Early gyroscopes were based on the inertia of a rotating rigid body. Later, vibratory and optical gyroscopes were subsequently developed. This paper is concerned with vibratory gyroscopes in which vibrations are usually excited and detected piezoelectrically or electrostatically. The literature on vibratory gyroscopes is numerous. Early references can be found in a few review articles [1,2,3] and Ph.D. dissertations [4,5]. Relatively recent ones are, e.g., [6,7,8,9,10], among which, [10] is a review on micromachined and nano gyroscopes.

Specifically, for piezoelectric vibratory gyroscopes based on flexural vibrations of thin beams [11], since piezoelectric coupling produces strains rather than curvatures, either a composite beam or some complicated electrode configuration is typically needed to excite and detect flexural motions of beams [11,12,13].

Recently, there has been a growing interest in the flexoelectric effect [14,15,16,17], with which, flexural motion in a homogeneous beam [18,19,20] or plate [21] can be excited or detected with only electrodes. In particular, flexoelectric beams have already been used as actuators or sensors in electromechanical devices [22,23]. This offers the possibility of flexoelectric angular rate sensors. In this paper, we propose a flexoelectric beam vibratory gyroscope that is original. The flexoelectric beam in the proposed gyroscope functions as both an actuator and a sensor at the same time through two pairs of electrodes and flexural vibrations in perpendicular directions. We demonstrate how the proposed gyroscope works through modeling. The basic three-dimensional theory of flexoelectricity is gathered in Section 2, from which, a one-dimensional model for flexural motions of the gyroscope is established in Section 3. A theoretical analysis and numerical results are presented in Section 4 and Section 5, respectively, to show the basic response of the beam when it is rotating about its axis. Finally, some conclusions are drawn in Section 6.

## 2. Theory of Flexoelectricity

The macroscopic theory of flexoelectricity [15,16,17] is summarized below for its notation. It is also the foundation for the one-dimensional (1-D) model to be developed in the next section. In Cartesian tensor notation [24], the field equations are

(1a) $$T_{ij,j}-\tau_{ijk,jk}+F_{i}=\rho {\ddot{u}}_{i},$$

(1b) $$D_{i,i}=0,$$

where **T** is the stress tensor, **τ** a higher-order stress, **F** the body force vector, *ρ* the mass density, which is a scalar, **u** the mechanical displacement vector, and **D** the electric displacement vector. A vector or tensor is written either in boldface or in component form with one index (vector) or more indices (tensor) [24]. A comma followed by an index denotes partial differentiation with respect to the coordinate associated with the index [24]. We limit ourselves to nonpiezoelectric materials. The constitutive relations accompanying Equation (1) describing material behaviors are

(2) $$\begin{array}{l} T_{ij}=C_{ijkl}S_{kl},\;\tau_{ijk}=-f_{lijk}E_{l},\\ D_{i}=\varepsilon_{ij}E_{j}+f_{ijkl}\eta_{jkl}, \end{array}$$

where **S** is the strain tensor, **E** the electric field vector, **η** the strain gradient tensor, $C_{ijkl}^{}$ the elastic stiffness tensor, $f_{ijkl}^{}$ the flexoelectric constants (tensor), and $\varepsilon_{ij}^{}$ the dielectric constants (tensor). **S**, **η**, and **E** are related to **u** and the scalar electric potential *ϕ* through

(3) $$S_{ij}=(u_{i,j}+u_{j,i})/2,\;\eta_{jkl}=S_{jk,l},\;E_{i}=-\varphi_{,i}.$$

## 3. One-Dimensional Equations for a Flexoelectric Beam in Bending Vibrations

**Figure 1:**
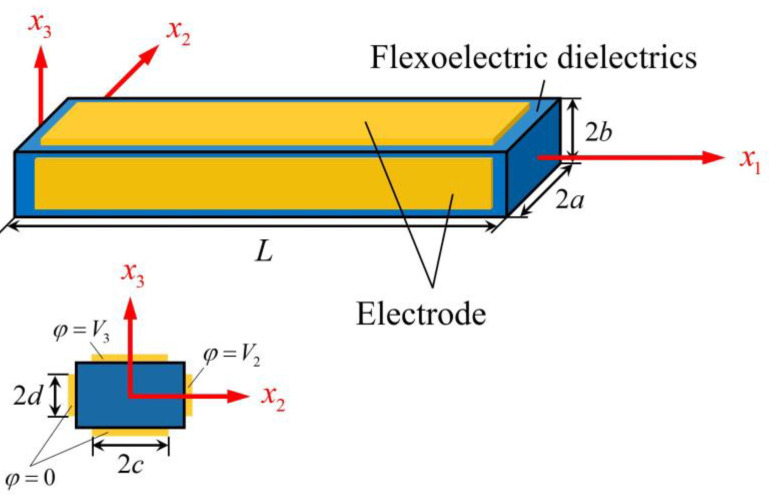
A thin flexoelectric beam and coordinate system whose origin is at the center of the left face.

Consider the thin flexoelectric beam in Figure 1. Its lateral surfaces are traction free and are electroded. The voltage across the two electrodes at *x*_2_ = ± *a* for actuation is *V*_2_(*t*), and that between the two electrodes at *x*_3_ = ± *b* for sensing is *V*_3_(*t*).

1-D equations for bending in the (*x*_1_,*x*_3_) plane were derived from Equations (1)–(3) in [20]. For the gryroscope application to be studied in the next section, we need to generalize the 1-D equations in [20] to the case of simultaneous bending in both of the the (*x*_1_,*x*_2_) and (*x*_1_,*x*_3_) planes. In this case, the displacement components are approximated by

(4) $$\begin{array}{l} u_{2}≅u_{2}^{}(x_{1},t),\;u_{3}≅u_{3}^{}(x_{1},t),\\ u_{1}≅-x_{2}u_{2,1}^{}-x_{3}u_{3,1}^{}, \end{array}$$

which produce the following axial strain and strain gradients:

(5) $$\begin{array}{l} S_{11}=-x_{2}u_{2,11}^{}-x_{3}u_{3,11}^{},\\ \eta_{112}=-u_{2,11}^{},\;\eta_{113}=-u_{3,11}^{}. \end{array}$$

Since the lateral surfaces of the beam are electroded and the electric potential are functions of time only on ideal electrodes that we assume, the electric field is approximated by

(6) $$E_{2}=-\frac{V_{2}}{2a},\;E_{3}=-\frac{V_{3}}{2b},\;E_{1}=0.$$

The equations for bending are obtained by integrating Equation (1a) with *i* = 2 and 3 over the beam cross section, which results in [20]

(7) $$\begin{array}{l} Q_{2,1}+f_{2}A=\rho A{\ddot{u}}_{2},\\ Q_{3,1}+f_{3}A=\rho A{\ddot{u}}_{3}, \end{array}$$

where *Q*_2_ and *Q*_3_ are the transverse shear forces in the *x*_2_ and *x*_3_ directions, respectively, and *A* = 4*ab* is the area of the beam cross section. The integration of the products of Equation (1a) with *x*_2_ or *x*_3_ over the beam cross section yields the following shear force-bending moment relation [20]:

(8) $$Q_{2}=M_{3,1},\;Q_{3}=M_{2,1}$$

where *M*_3_ and *M*_2_ are moments associated with bending in the (*x*_1_,*x*_2_) and (*x*_1_,*x*_3_) planes, respectively. For thin beams, the rotatory inertia is neglected. The 1-D constitutive relations are obtained by integrating the relevant equations in Equation (2) and their products with *x*_2_ or *x*_3_ over a cross section. The results are

(9a) $$M_{3}=-\bar{c}B_{2}u_{2,11}-f_{3113}AE_{2},$$

(9b) $$M_{2}=-\bar{c}B_{3}u_{3,11}-f_{3113}AE_{3},$$

(9c) $$D_{2}^{\left(0\right)}=\varepsilon_{11}AE_{2}-f_{3113}Au_{2,11},$$

(9d) $$D_{3}^{\left(0\right)}=\varepsilon_{11}AE_{3}-f_{3113}Au_{3,11},$$

where

(10) $$\begin{array}{l} \bar{c}=\frac{\left(c_{1111}-c_{1122})(c_{1111}+2c_{1122}\right)}{c_{1111}+c_{1122}},\\ B_{2}=\int_{A}x_{2}^{2}dA,\;B_{3}=\int_{A}x_{3}^{2}dA. \end{array}$$

$D_{2}^{\left(0\right)}$ and $D_{3}^{\left(0\right)}$ are needed to calculate the charge on the electrodes. Substitutions from Equations (8) and (9), we can write Equation (7) as two equations for *u*_1_ and *u*_2_:

(11) $$\begin{array}{l} -\bar{c}B_{2}u_{2,1111}+F_{2}A=\rho A{\ddot{u}}_{2},\\ -\bar{c}B_{3}u_{3,1111}+F_{3}A=\rho A{\ddot{u}}_{3}. \end{array}$$

## 4. Analysis of a Flexoelectric Gyroscope

When the beam in Figure 1 is used as a gyroscope, it is rotating about the *x*_1_ axis with an angular rate Ω that is to be measured. We fixed the coordinate system to the rotating beam. In the rotating coordinate system, the effects of the centripetal and Coriolis accelerations can be taken into consideration through the following effective forces:

(12) $$\begin{array}{l} F_{2}=-\rho (-2\Omega {\dot{u}}_{3}^{}-\Omega^{2}u_{2}^{}),\\ F_{3}=-\rho (2\Omega {\dot{u}}_{2}^{}-\Omega^{2}u_{3}^{}). \end{array}$$

*V*_2_ is the known applied voltage that drives the beam into bending with *u*_2_. The effective Coriolis force then drives the beam into bending with *u*_3_, which produces *V*_3_, which is unknown. The charge on the electrode at *x*_3_ = *b* is given by

(13) $$\begin{array}{l} Q^{e}=2c\int_{0}^{L}-{D_{3}|}_{x_{3}=b}dx_{1}\\ \;=\frac{2cL\varepsilon_{11}V_{3}}{2b}+2cf_{3113}[u_{3,1}(L)-u_{3,1}(0)], \end{array}$$

where Equation (9d) has been used. The current flowing out of this electrode is given by

(14) $$I_{3}=-{\dot{Q}}^{e}.$$

Consider time-harmonic motions with the following complex notation

(15) (V2,V3,u2,u3,Qe,I3)=Re{(V¯2,V¯3,U2,U3,Q¯e,I¯3)exp(iωt},

where ${\bar{V}}_{2}$, ${\bar{V}}_{3}$, *U*_2_, *U*_3_, ${\bar{Q}}^{e}$, and ${\bar{I}}_{3}$ are the complex amplitudes of *V*_2_, *V*_3_, *u*_2_, *u*_3_, *Q^e^*, and *I*_3_. *i* is the imaginary unit. ω is the time-harmonic frequency. The electrodes at *x*_3_ = ± *b* are connected by a circuit whose impedance is *Z* in harmonic motions, which provides the following circuit equation:

(16) $${\bar{I}}_{3}=\;{\bar{V}}_{3}/Z.$$

The substitution of Equations (12)–(15) into Equations (11) and (16) results in the following three equations for *U*_2_, *U*_3_, and $\;{\bar{V}}_{3}$:

(17a) $$-\bar{c}B_{2}U_{2,1111}+\rho A(\omega^{2}U_{2}+i\omega 2\Omega U_{3}+\Omega^{2}U_{2})=0,$$

(17b) $$-\bar{c}B_{3}U_{3,1111}+\rho A(\omega^{2}U_{3}-i\omega 2\Omega U_{2}+\Omega^{2}U_{3})=0,$$

(17c) $$\frac{{\bar{V}}_{3}}{Z}=-2i\omega c\{\frac{L\varepsilon_{11}{\bar{V}}_{3}}{2b}+f_{3113}[U_{3,1}(L)-U_{3,1}(0)]\}.$$

Specifically, consider a simply supported beam with the following boundary conditions:

(18) $$\begin{array}{l} u_{2}(0,t)=u_{3}(0,t)=u_{2}(L,t)=u_{3}(L,t)=0,\\ M_{2}(0,t)=M_{3}(0,t)=M_{2}(L,t)=M_{3}(L,t)=0. \end{array}$$

Equation (18) represents the simplest and most basic mounting of a beam, which was used in the first piezoelectric vibratory gyroscope [11]. The purpose of the present paper is to show that a vibrating flexoelectric beam can also operate as a gyroscope. Other mountings, such as a cantilever, can also be used, which changes the mathematical analysis but not the mechanism of the device. Therefore, other boundary conditions are not pursued here.

Equation (17a,b) form a system of linear ordinary differential equations. We look for its solution in the following form:

(19) $$U_{2}={\bar{U}}_{2}\exp(kx_{1}),\;U_{3}={\bar{U}}_{3}\exp(kx_{1})$$

where *k* is undetermined. The substitution of Equation (19) into Equation (17a,b) gives two linear homogeneous algebraic equations:

(20) $$\left[\begin{array}{cc} \rho A(\omega^{2}+\Omega^{2})-\bar{c}B_{2}k^{4} & 2\rho Ai\omega \Omega \\ -2\rho Ai\omega \Omega & \rho A(\omega^{2}+\Omega^{2})-\bar{c}B_{3}k^{4} \end{array}\right]\left\{\begin{array}{l} {\bar{U}}_{2}\\ {\bar{U}}_{3} \end{array}\right\}=\left\{\begin{array}{l} 0\\ 0 \end{array}\right\}.$$

For nontrivial solutions, the determinant of the coefficient matrix has to vanish, i.e.,

(21) $${\left(\bar{c}\right)}^{2}B_{2}B_{3}k^{8}-\rho A(\omega^{2}+\Omega^{2})\bar{c}(B_{2}+B_{3})k^{4}+{\left[\rho A(\omega^{2}+\Omega^{2})\right]}^{2}+{\left(2\rho Ai\omega \Omega \right)}^{2}=0.$$

Equation (21) is a polynomial equation for *k*. We denote its eight roots by *k*^(*N*)^, where *N* = 1, 2, …, 8. The corresponding nontrivial solutions of *U*_2_ and *U*_3_ are denoted by

(22) $$\left\{\begin{array}{l} U_{2}^{}\\ U_{3}^{} \end{array}\right\}=\left\{\begin{array}{l} 2\rho Ai\omega \Omega \\ -[\rho A(\omega^{2}+\Omega^{2})-\bar{c}B_{2}{\left(k^{\left(N\right)}\right)}^{4}] \end{array}\right\}.$$

Then, the general solution of Equation (17a,b) can be written as

(23) $$\begin{array}{l} U_{2}=\sum_{N=1}^{8}\left(2\rho Ai\omega \Omega ){\bar{U}}^{\left(N\right)}\exp(k^{\left(N\right)}x_{1}\right),\\ U_{3}=-\sum_{N=1}^{8}[\rho A(\omega^{2}+\Omega^{2})-\bar{c}B_{2}{\left(k^{\left(N\right)}\right)}^{4}]{\bar{U}}^{\left(N\right)}\exp(k^{\left(N\right)}x_{1}), \end{array}$$

where ${\bar{U}}^{\left(N\right)}$ are undetermined constants. The substitution of Equation (23) into Equations (17c) and (18) yields nine linear algebraic equations for ${\bar{U}}^{\left(N\right)}$ and ${\bar{V}}_{3}$. These equations are solved on a computer using MATLAB R2021a (Xi’an, China).

## 5. Numerical Results and Discussion

As a numerical example, consider a ceramic beam of BaTiO_3_ that is not poled and hence is nonpiezoelectric. The relevant material constants are *C*_11_ = 166 GPa, *C*_12_ = 77 GPa, *C*_13_ = 78 GPa, *C*_33_ = 162 GPa, *C*_44_ = 43 GPa, *ϵ*_33_ *=* *ϵ*_22_ = 12.6 × 10^−9^ C^2^/(N∙m^2^), and *f*_3113_ = 10^−6^ N/C. The elastic and dielectric constants are from [25]. The flexoelectric constant is from [18,26]. Examples of other materials that have been used for micro-beams are zinc oxide, barium sodium niobate, barium titanate [27], and strontium titanate [28], which, when unpoled, may be considered for flexoelectric gyroscope applications. In [29], a micro-beam of BaTiO_3_ with dimensions of 1.5, 3.2, and 11 μm was fabricated for experimental investigation. For our modeling analysis with the goal of demonstrating the basic operation of the gyroscope, the geometric parameters were chosen to be *a* = *b* = 5 μm, *c* = *d* = *a*/2, and *L* = 200 μm. Material damping is described by complex elastic constants *C_pq_*(1 + i/*Q*) with *Q* = 10^2^. The amplitude of the driving voltage is *V*_2_ = 100 volts. *Z* = ∞ is used for the open circuit output voltage. Ω = 3.6 × 10^4^ rad/s, which is much smaller than (approximately 1%) the first resonance frequency of the beam, which is 3.6 × 10^6^ rad/s. Some of these parameters may be varied separately below. We introduced *Z*_2_ as a unit for *Z*:

(24) $$Z_{2}=\frac{1}{2i\omega cC_{2}},\;C_{2}=\frac{L\varepsilon_{11}}{2b}.$$

**Figure 2:**
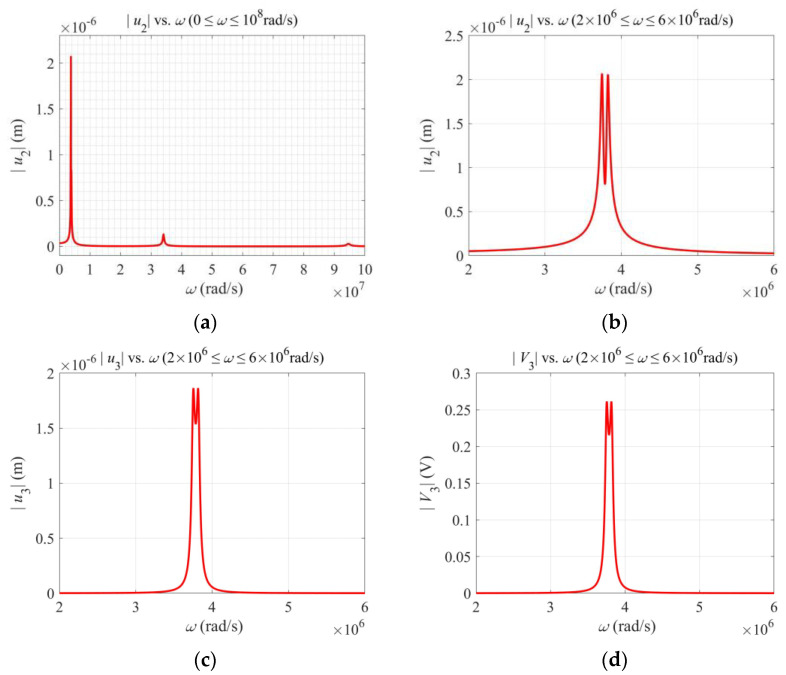
Behavior near the first resonance. (**a**) Primary motion showing resonances. (**b**) Primary motion near the first resonance. (**c**) Secondary motion near the first resonance. (**d**) Output voltage near the first resonance.

Figure 2a shows $\left|u_{2}(L/2)\right|$ versus the driving frequency *ω* with three resonances. The third one is barely visible. For gyroscope application, we are mainly interested in the first resonance. $\left|u_{2}(L/2)\right|$, $\left|u_{3}(L/2)\right|$ and the output voltage |*V*_3_| near the first resonance are shown in Figure 2b–d, respectively. *u*_2_ is driven by the applied *V*_2_ through flexoelectric coupling and is called the primary motion. *u*_3_ is due to the Coriolis force associated with Ω and is called the secondary motion. *V*_3_ is produced by *u*_3_ through flexoelectric coupling. They all assume double-peak resonances because of flexural vibrations in both directions, which is typical for vibratory piezoelectric gyroscopes.

**Figure 3:**
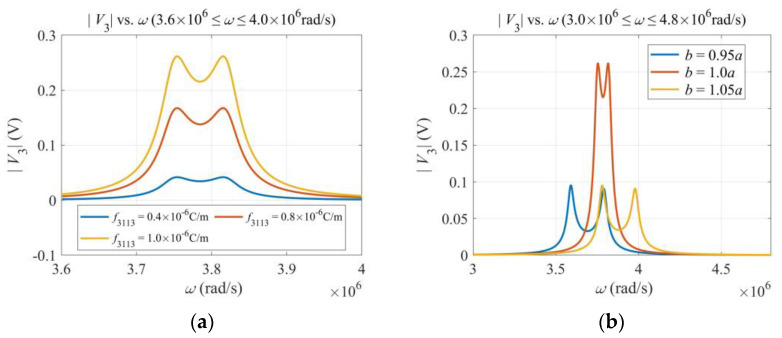

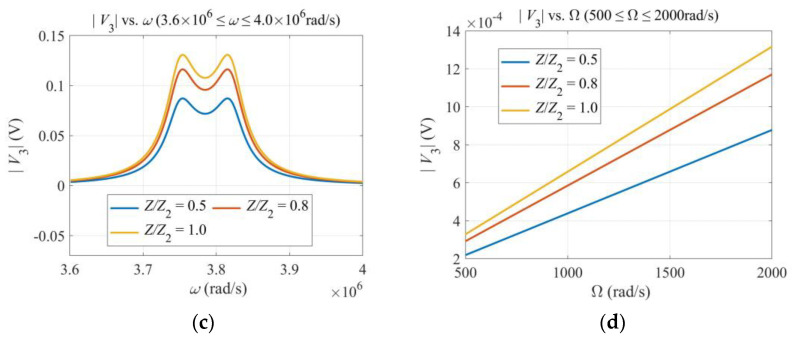
Effects of various parameters. (**a**) Flexoelectric coefficient. (**b**) Dimensions of the cross section. (**c**) Impedance of the output circuit. (**d**) Angular rate Ω. ω = 3.7 × 106 rad/s.

Figure 3 shows the effects of various parameters on the output voltage near the first resonance. Figure 3a shows that a larger flexoelectric coefficient leads to a higher output, which is as expected. Figure 3b shows that the output voltage drops when the cross section deviates somewhat from a square. This is because, for a beam with a cross section not close to a square, the resonance frequencies of flexural vibrations in the *x*_2_ and *x*_3_ directions are not close. Hence, the gyroscope is not working in the optimal condition (the so-called double-resonant condition). Figure 3c shows that, when the impedance of the output circuit increases, the output voltage increases too. At the same time, the output current decreases correspondingly. Figure 3d shows that the output voltage is linear in Ω when Ω is small, which is ideal for angular rate sensing. For large values of Ω, the linearity is lost because Ω appears in Equation (17) in a complicated and nonlinear way.

**Figure 4:**
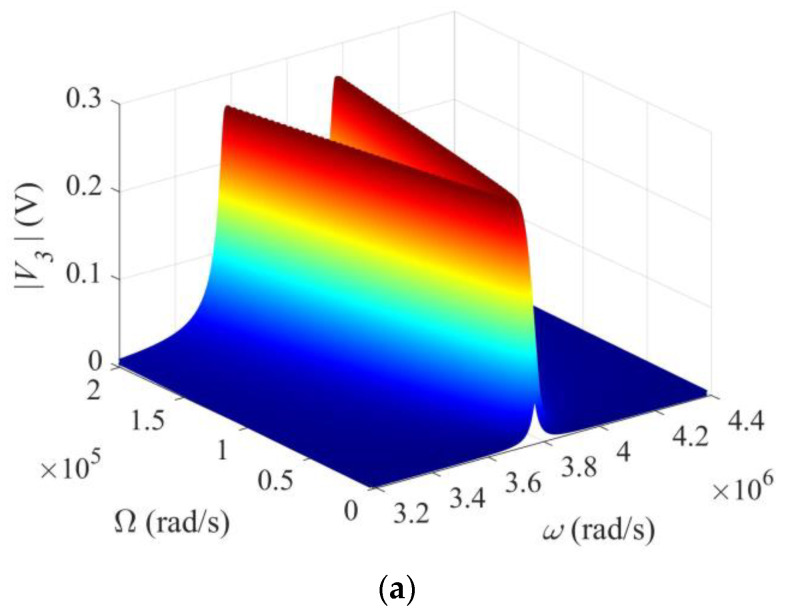

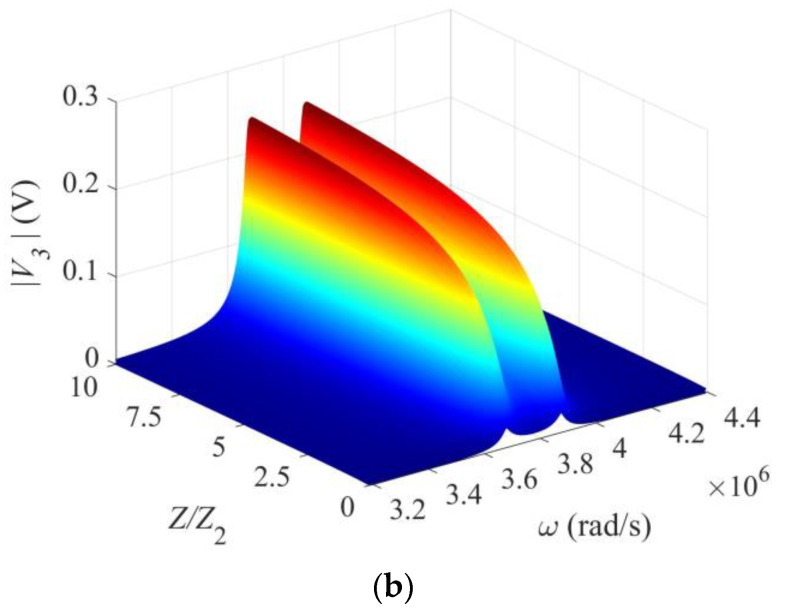
Three-dimensional views of the output voltage. (**a**) V3 versus ω and Ω. Z = ∞. (**b**) V3 versus ω and Z. Ω = 105 rad/s.

The output signal *V*_3_ for detecting Ω depends on several physical and geometric parameters; in particular, the driving frequency *ω* and the impedance *Z* of the output circuit as shown in Figure 2 and Figure 3, where *ω* and *Z* were varied one at a time. For a more comprehensive understanding of the behavior of the gyroscope, we plot *V*_3_ versus ω and Ω together in Figure 4a, and *V*_3_ versus ω and *Z* in Figure 4b, respectively. The curves in Figure 2 and Figure 3 are formed by intersections of the surfaces in Figure 4 with different vertical planes. It can be seen from Figure 4a that, when Ω is fixed, there are two peaks as ω varies. The two peak values increase with Ω monotonically when Ω is small and saturate when Ω is large. The distance between the two peaks also increases with Ω. In Figure 4b, when *Z* is fixed, there are two peaks as ω varies. The two peak values increase with *Z* monotonically. When *Z* is small, the output circuit is nearly shorted, with a small *V*_3_. When *Z* is large, the output circuit is nearly open, with a large and saturated *V*_3_. These agree with Figure 3c,d.

## 6. Conclusions

It is shown theoretically that a micro-beam in flexural vibrations excited and detected flexoelectrically can be used to make a gyroscope to detect an angular rate. Compared to conventional piezoelectric beam gyroscopes, the flexoelectric beam gyroscope proposed has a simpler structure or electrode configuration. The one-dimensional model developed is effective in describing the basic behaviors of the beam flexoelectric gyroscope.

## Data Availability

Not applicable.
